# Mechanism of Action of the Tumor Vessel Targeting Agent NGR-hTNF: Role of Both NGR Peptide and hTNF in Cell Binding and Signaling

**DOI:** 10.3390/ijms20184511

**Published:** 2019-09-12

**Authors:** Barbara Valentinis, Simona Porcellini, Claudia Asperti, Manuela Cota, Dan Zhou, Paola Di Matteo, Gianpiero Garau, Chiara Zucchelli, Nilla Roberta Avanzi, Gian Paolo Rizzardi, Massimo Degano, Giovanna Musco, Catia Traversari

**Affiliations:** 1 MolMed SpA, 20132 Milano, Italy; barbara.valentinis@molmed.com (B.V.); simona.porcellini@molmed.com (S.P.); claudia.asperti@molmed.com (C.A.); manuela.cota@molmed.com (M.C.); dan.zhou@molmed.com (D.Z.); paola.di_matteo@novartis.com (P.D.M.); paolo.rizzardi@altheiascience.com (G.P.R.); 2 Biocrystallography Unit, Division of Immunology, Transplantation and Infectious Diseases, IRCCS San Raffaele Scientific Institute, 20132 Milano, Italy; Gianpiero.Garau@iit.it (G.G.); degano.massimo@hsr.it (M.D.); 3 Biomolecular NMR Unit, Division of Genetics and Cell Biology, IRCCS San Raffaele Scientific Institute, 20132 Milano, Italy; zucchelli.chiara@hsr.it (C.Z.); musco.giovanna@hsr.it (G.M.); 4 Biotechnology Department, Nerviano Medical Sciences S.r.l., 20014 Nerviano, Italy; Nilla.Avanzi@nervianoms.com

**Keywords:** NGR peptide, vascular targeting, TNF, CD13, signal transduction

## Abstract

NGR-hTNF is a therapeutic agent for a solid tumor that specifically targets angiogenic tumor blood vessels, through the NGR motif. Its activity has been assessed in several clinical studies encompassing tumors of different histological types. The drug’s activity is based on an improved permeabilization of newly formed tumor vasculature, which favors intratumor penetration of chemotherapeutic agents and leukocyte trafficking. This work investigated the binding and the signaling properties of the NGR-hTNF, to elucidate its mechanism of action. The crystal structure of NGR-hTNF and modeling of its interaction with TNFR suggested that the NGR region is available for binding to a specific receptor. Using 2D TR-NOESY experiments, this study confirmed that the NGR-peptides binds to a specific CD13 isoform, whose expression is restricted to tumor vasculature cells, and to some tumor cell lines. The interaction between hTNF or NGR-hTNF with immobilized TNFRs showed similar kinetic parameters, whereas the competition experiments performed on the cells expressing both TNFR and CD13 showed that NGR-hTNF had a higher binding affinity than hTNF. The analysis of the NGR-hTNF-triggered signal transduction events showed a specific impairment in the activation of pro-survival pathways (Ras, Erk and Akt), compared to hTNF. Since a signaling pattern identical to NGR-hTNF was obtained with hTNF and NGR-sequence given as distinct molecules, the inhibition observed on the survival pathways was presumably due to a direct effect of the NGR-CD13 engagement on the TNFR signaling pathway. The reduced activation of the pro survival pathways induced by NGR-hTNF correlated with the increased caspases activation and reduced cell survival. This study demonstrates that the binding of the NGR-motif to CD13 determines not only the homing of NGR-hTNF to tumor vessels, but also the increase in its antiangiogenic activity.

## 1. Introduction

The recombinant cytokine NGR-TNF, carrying the CNGRC tumor homing peptide [1], has shown a higher anti-tumor activity than the corresponding wild type cytokine in animal models [2], thus allowing its use at low, not toxic concentrations. This enhanced efficacy is due to the CNGRC motif that, through the binding to an isoform of the aminopeptidase N (also called CD13), selectively expressed by angiogenic vessels, increases the delivery of the tagged cytokine at the tumor site [3]. The in vivo effects of NGR-TNF are multiple and include an alteration of the endothelial barrier function, increased tumor permeability [4,5], and immunological effects [6]. It has been demonstrated that NGR-TNF induces apoptosis of tumor and endothelial cells in vivo [7,8], and that it can promote, through the involvement of the immune system, vessel normalization and an immune-supportive microenvironment, which ultimately results in reduced tumor growth [8]. NGR-hTNF has been administered as an anti-cancer drug alone or in combination with chemotherapy, in several clinical studies encompassing tumors of different histological types (reviewed in [9]). Among others, significant results have been recently reported in small cell lung cancer [10], in mesothelioma patients who rapidly progressed after the first-line treatment [11], and in brain lymphomas [5]. The efficacy of new therapeutic strategies combining NGR-hTNF treatment with active or adoptive immunotherapies were also investigated, at a preclinical and clinical level [12,13]. 

It has been demonstrated that both the TNF-receptor (TNFR) and CD13 have a role in NGR-hTNF activity [2]: The first mediating the cytokine effects; the second allowing the vascular targeting. TNFR, engaged by hTNF, activates several pro-death/stress (caspases, JNK, and p38), and pro-survival (NFκB, ERK, and Akt) signal-transduction pathways in several cells (reviewed in [14,15]), including endothelial cells [16,17]. The final cellular response is the result of a complex and finely controlled interaction of multiples pathways, and depends on the hTNF dose, the interaction with other stimuli such as growth factors, the adhesion to extracellular matrix, and the cell type [18,19,20]. hTNF-induced cell death occurs by both regulated necrosis and apoptosis [15], and apoptosis is in turn mediated by autolytic activation of the initiator caspase-8, which activates the effector caspase-3 [21,22]. Caspases activation and/or activity is subjected to a tight regulation by posttranslational modifications and the interaction with modulatory proteins (reviewed in [23]). Indeed, caspases are inhibited by kinases such as Erk and Akt [24,25], and by NFκ-B, which induces transcriptional activation of the suppressors IAP and FLIP [26,27,28]. 

The second receptor involved in the NGR-hTNF activity is the CD13 isoform selectively expressed on the tumor endothelial cells [8,29,30], that mediate the binding to the CNGRC motif. CD13, also expressed in a few normal tissues, several tumors and myeloid cells [30,31], is a multiple function ectoenzyme with a role in viral binding, antigen presentation, angiogenesis, and in cell adhesion, motility and invasion (reviewed in [32]). Interestingly, some of the CD13 functions are mediated by the direct triggering of a signal transduction pathway [33,34], or by the modulation of the signals initiated by other receptors [35,36]. As CD13 does not possess intrinsic catalytic activity, it may exert its effects through the association with auxiliary proteins [34,37], or by influencing the plasma membrane protein organization [36]. 

Despite the large body of evidence supporting the vascular targeting properties of the NGR-hTNF and its vascular and antitumor effects, a structural and biochemical characterization of the interaction between the recombinant cytokine and its receptors, TNFR and CD13, is still lacking. This study showed that both the CNGRC peptide and hTNF components have a role in cell binding and signaling, providing new evidence on the mechanism of action of NGR-hTNF, in support of its antitumor activities. 

## 2. Results

### 2.1. Crystal Structure of NGR-hTNF

Single crystal X-ray diffraction was used to assess whether the CNGRC peptide induces a preferred conformation of the N-terminus of hTNF in the NGR-hTNF molecule (Table 1). 

**Figure 1:**
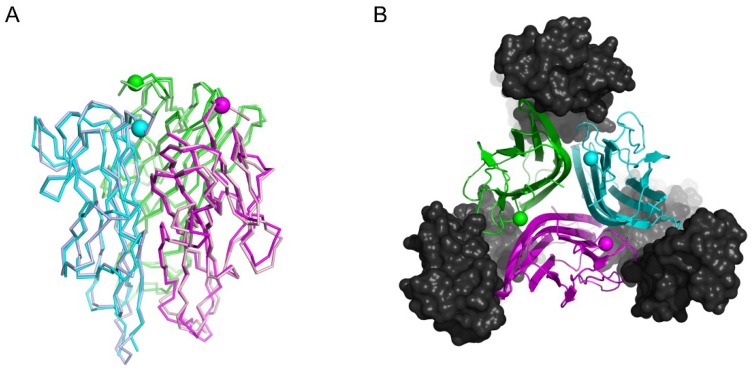
Conformational features of NGR-hTNF monomer. (**A**) Crystal structure of the NGR-hTNF trimer (PDB ID 6RMJ, with individual chains of the trimer colored darker shades of blue, pink, and green) superimposed with hTNF (PDB ID 1TNF, individual chains colored in lighter shades), underscoring the high similarity between the two proteins (**B**) Superposition of the NGR-hTNF structure (in color) onto the crystal structure of the LTα-TNFR1 complex (black, PDB ID 1TNR). In both panels, the first ordered residue in the crystal structure of NGR-hTNF in each chain, Ser9, is shown as a sphere. The N-terminal extension can extend into the solvent without interfering with receptor binding.

The 2.65 Å crystal structure of NGR-hTNF is substantially identical to that observed in the absence of the N-terminal extension [38], with a rmsd of 0.81 Å for 373 Cα and 0.95 Å for 2701 total atoms in the trimeric arrangement (Figure 1A). The N-terminal region of the mature hTNFα (amino acids 1–8) and the CNGRCG hexapeptide could not be traced due to the lack of a significant continuous electron density in the difference maps. These findings indicate that the N-terminus of the NGR-hTNF molecule does not interact stably with the cytokine, is endowed with very high flexibility and can thus attain many possible orientations. Hence, the crystal structure of NGR-hTNF demonstrates that the targeting peptide in NGR-hTNF does not stabilize a different, specific conformation of the cytokine, and remains available for the interaction with accessory receptors such as CD13. This study superimposed the newly determined structure of NGR-hTNF onto the crystal structure of the lymphotoxin α-TNFR1 complex [39] to model a possible geometry of interaction (Figure 1B). In this model, the N-terminal region of NGR-hTNF is positioned between the three TNFR1 molecules, allowing the preceding peptide to extend in the solvent and thus interact with other receptors. These findings suggest that the N-terminal fusion peptide does not hinder the interactions with the TNFR1, and could be available for binding to specific molecular determinants on the cell surface, thus modulating the TNF activity.

### 2.2. In Vitro Characterization of the Binding Properties of the CNGRC Peptide

**Figure 2:**
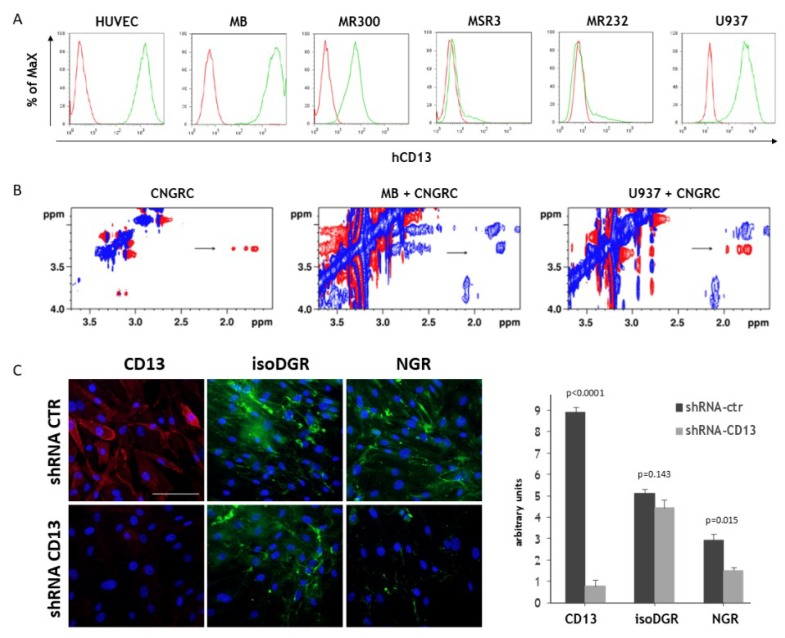
CD13 expression and NGR-binding properties in different cell lines. (**A**) Flow cytometry analysis of CD13 expression (green histograms) in the indicated cell lines. Red histograms represent isotype control. (**B**) Representative 2D TR-NOESY spectra are shown. Left spectrum (tmix = 600 ms): free CNGRC with positive NOEs (red peaks). Middle spectrum (tmix = 100 ms): CNGRC, in the presence of MB cells, transiently adopts, during binding to the CD13, the tumbling time of the receptor and transfer the negative NOE of the protein complex to the population of the free molecule (blue peaks). At longer mixing time (600 ms) the signal has decayed (Supplementary Figure S1B). Right spectrum (tmix = 600 ms): CNGRC, in the presence of U937 cells, does not bind to the receptor, thus its NOE cross peaks are positive (red peaks). The arrows indicate the cross-peaks deriving from correlations of the arginine δ/γ and δ/β protons of the CNGRC peptide. (**C**) Binding of NGR-biotin (green) to target cells (MB) is prevented by CD13 silencing with shRNA, whereas isoDGR-biotin (green), an unrelated integrin-specific peptide, does not. The down modulation of the CD13 receptor is confirmed by staining with the anti-CD13 Ab (red). On the left panel, a representative experiment is shown (Scale Bar, 50 µm). On the right panel, the quantification of the binding is reported (media and SE of three independent experiments).

The CNGRC motif is known to bind a CD13 isoform expressed by the cells lining angiogenic vessels [29,30,40] and some cancer cells of neoplastic human tissues [30]. CNGRC binding also occurs to human umbilical vein endothelial cells (HUVEC) [41], but not to myeloid CD13-expressing cells [30]. To investigate the binding capability of NGR-hTNF, the cell lines with different levels of CD13 expression were used, as determined by FACS analysis (Figure 2A). HUVEC, mesangioblasts (MB) showing the typical markers of pericytes [42], the lung carcinoma MR300 cell line, and the myelomonocytic U937 cells were CD13 positive. Two tumor cell lines, MSR3 and MR232, that did not express CD13, were used as a negative control. 

The interaction between a cyclic CNGRC peptide and these cell lines was investigated by the 2D Transferred Nuclear Overhauser effect (TR-NOE) experiments, using intact cells (representative spectra are reported in Figure 2B). The TR-NOE relies on the rapid and efficient transfer of spectroscopic characteristics between the free and the bound state of a ligand [43]. When the ligand is free in solution, its Nuclear Overhauser Effect Spectroscopy (NOESY) spectrum shows positive NOE correlations (opposite sign with respect to the diagonal), as shown in Figure 2B (left spectrum, read peaks). In the presence of the cells, if the ligand binds to the receptor, it transiently adopts the tumbling time of the receptor attached to the cell and can thus transfer the negative NOE (same sign with respect to the diagonal) of the protein complex to the population of the free molecule (Figure 2B, middle spectrum, blue peaks). If the ligand does not bind, the NOE cross-peaks outside the diagonal maintain the positive sign (Figure 2B, right spectrum, read peaks). NOESY spectra acquired on CNGRC in the presence of the different cell lines studied are summarized in Supplementary Figure S1A. The results indicate that, as expected, CNGRC binds to HUVEC, MB, and the CD13 positive tumor cell lines (MR300), but not to MSR3 cells, which do not express CD13. Interestingly, CNGRC in the presence of U937 cells generated spectra with positive NOE effects, thus indicating that the peptide remains free in solution despite the expression of CD13 on the cell surface. This result, in agreement with the fact that NGR-hTNF and hTNF have similar cytotoxic activity on U937 cells [2], shows that U937 cells express a CD13 isoform that is not recognized by the CNGRC sequence. The negative control peptides, (CARGC or CDGRC) that do not bind CD13 also generated, as expected, positive NOE effects (Supplementary Figure S1A). The pattern of the interaction between the CNGRC motif and the analyzed cell lines was confirmed by staining with the CNGRCG-hTNF(1–11) peptide, (corresponding to the 17 N-terminal aminoacids of the NGR-hTNF), biotinylated (NGR-biotin) or conjugated to quantum dots (NGR-Qd). MB (Figure 2C, ctr lane), HUVEC [41], and MR300 cells (data not shown) were stained by the CNGRC peptide, whereas U937 and GR4-mel cells were not stained (data not shown).

Finally, to prove the specificity of the ligand–receptor interaction in living cells, the MB cells were transduced with a lentiviral vector coding for a CD13-specific shRNA, and the binding to the NGR-biotin was examined. As expected, silencing of CD13 strongly reduced the binding of both anti-CD13 mAb and NGR-biotin to shCD13-cells (Figure 2 C), whereas the binding of isoDGR-biotin, that is specific for αvβ3-integrin [44], was not affected. Altogether, these results demonstrate that the expression of CD13 is necessary but not sufficient for the binding of CNGRC-containing peptides and confirm that the in vitro expression of the CNGRC-binder isoform of the CD13 is restricted to the cells that form the tumor vasculature (i.e., endothelial cells and pericytes) and to some tumor cell lines.

### 2.3. In Vitro Characterization of the Binding Properties of NGR-hTNF

**Figure 3:**
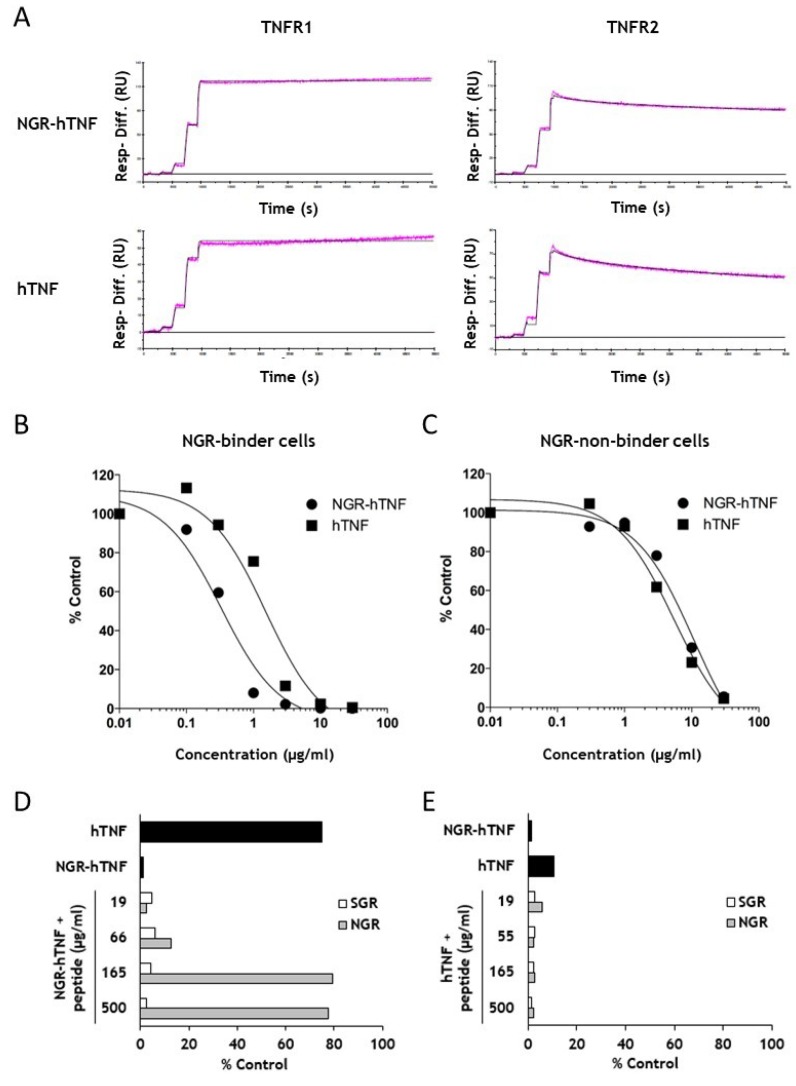
In vitro characterization of the binding properties of NGR-hTNF. (**A**) Sensograms of hTNF and NGR-hTNF kinetic titration on TNFR1 and TNFR2 (**B**) NGR-hTNF and hTNF efficiently compete the binding of hTNF-Fc in a semi-quantitative manner, but NGR-hTNF competes the hTNF-Fc binding to a greater extent than hTNF in CNGRC-binder cells (MR300). (**C**) NGR-hTNF and hTNF compete at the same extent the binding of hTNF-Fc to CNGRC-non-binder cells (GR4-mel). (**D**) On NGR-binder cells (MR300), the NGR-hTNF competition of the hTNF-Fc binding (black bar) is completely reverted by increasing concentrations of free CNGRC peptide (gray bars). As a control, competition with the unrelated CSGRC peptide is shown (white bars). (**E**) On NGR-binder cells (MR300), the competition exerted by hTNF on the hTNF-Fc binding (black bar) is not reverted by the addition of free CNGRC peptide (gray bars). As a control, competition with the unrelated CSGRC peptide is shown (white bars). The black bars in (**D**,**E**) indicates hTNF-Fc binding in the presence of NGR-hTNF or hTNF, expressed as percentage of control (i.e., hTNF-Fc alone).

To verify whether the presence of the CNGRC sequence modifies the interaction of the NGR-hTNF with hTNF receptors (TNFR1 and TNFR2), a titration kinetic analysis was performed using surface plasmon resonance (Figure 3A). The shape of the sensorgrams reflects a similar behavior of hTNF and NGR-hTNF in the binding of the receptors, with both cytokines showing a stronger binding for TNFR1 than for TNFR2. Then, the kinetic and thermodynamic parameters (k_on_, k_off_ and K_D_) of the interaction between hTNF or NGR-hTNF with TNFRs were determined. A complex interaction stoichiometry is expected, because the active form of hTNF or NGR-hTNF is a trimer that binds to three receptor molecules. Indeed, it was confirmed by stoichiometry evaluation that the hTNF or NGR-hTNF interaction with TNFRs is comprised between 1:2 and 1:3 at all the receptor densities tested (Supplementary Figure S2, and data not shown). The kinetic parameters were determined with a standard kinetic approach, which allows to fit the data with different models (1:1, 1:2 and 1:3). Based on chi^2^ statistics, it was confirmed that the results might fit with a complex bivalent or trivalent model, without the possibility of discriminating between the two. The results of the analyses are reported in Table 2.

The comparable K_D_ values were observed for hTNF and NGR-hTNF in the binding of both TNFR1 and TNFR2 receptors. In addition, the dissociation kinetics were analyzed with a bi-exponential decay equation that revealed a fast and a slow dissociating component, and confirmed the absence of differences between hTNF and NGR-hTNF dissociation kinetic from TNFRs (Supplementary Figure S3). In agreement with the literature, TNFR1 and TNFR2 showed a different dissociation kinetic, with k_off_ values for TNFR2 faster with respect to TNFR1. Taken together, these results demonstrate that the addition of the CNGRC peptide at the N-terminal does not modify the interaction of hTNF with immobilized TNFR1 and TNFR2. These results are consistent with the determined NGR-hTNF structure showing that the N-terminal region is flexible and does not modify the hTNF structure. Next, it was asked whether the concomitant presence of TNFRs and CD13 on the cell membrane could alter the interaction of hTNF or NGR-hTNF with the target cells. The adherent CNGRC-binder and non-binder cells were stained with a recombinant tagged hTNF-Fc protein alone or in the presence of an increasing amount of unlabeled hTNF or NGR-hTNF. hTNF and NGR-hTNF displaced hTNF-Fc binding on CNGRC-non-binder cells with the same efficiency (Figure 3C). On the contrary, a higher amount of hTNF than NGR-hTNF are required to obtain the same displacement of hTNF-Fc from CNGRC-binder cells (Figure 3B), indicating higher affinity of NGR-hTNF for TNFRs (NGR-hTNF IC_50_ ≈ 5 nM versus hTNF IC_50_ ≈20 nM). This result suggests that the CNGRC component of the NGR-hTNF might be responsible for the observed increase in TNFR affinity. This hypothesis was confirmed by the observation that in the presence of an increasing amount of free CNGRC peptide, the hTNF-Fc displacement mediated by NGR-hTNF reverted to the level obtained with hTNF (Figure 3D). No effects were observed with the free CNGRC peptide on the hTNF-Fc displacement mediated by hTNF (Figure 3E). Taken together, these results demonstrate that the interaction of hTNF with its receptors is not directly affected by the presence of the CNGRC peptide, but the latter increases the binding affinity of NGR-hTNF on the target cells expressing CD13.

### 2.4. In Vivo Characterization of the Binding Properties of the CNGRC Peptide

**Figure 4:**
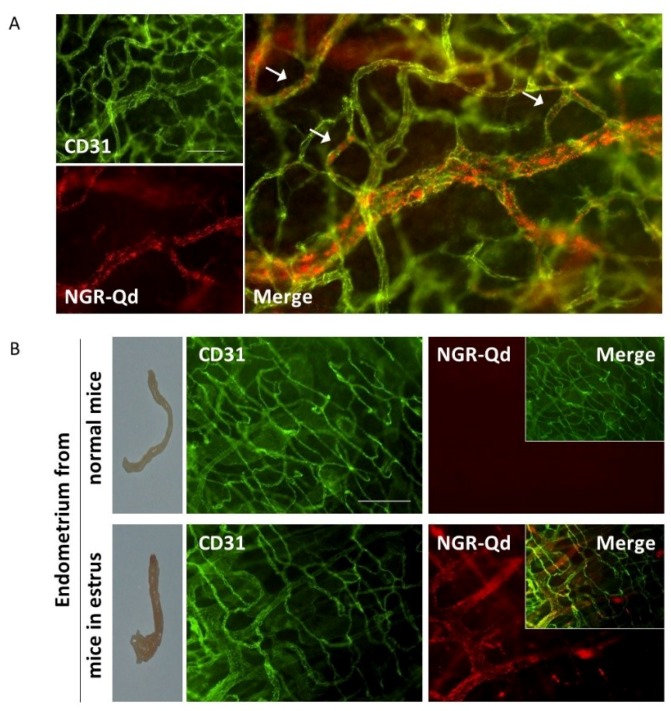
In vivo characterization of the binding properties of the CNGRC peptide. (**A**) In vivo-administered NGR-Qd into CT26 tumor-bearing mice specifically binds to tumor associated vessels. NGR-Qd (red) co-localizes onto tumor blood vessels of a murine colon carcinoma, stained by anti-CD31 Ab (green). Arrows show the staining of smaller vessels. Whole-mount histology with scale bar, 100 µm. (**B**) NGR-Qd (red) binds to the angiogenic endometrium from mice in estrus (lower panels) but not to the endometrium from mice not in estrus (upper panels). CD31 was used to stain blood vessels (green). Scale Bar, 200 µm.

To characterize the binding properties of the CNGRC motif in vivo, NGR-Qd were injected into CT26 tumor-bearing mice, in mice in estrus and in the respective normal counterparts. NGR-Qds localized along the vessels in the tumor (Figure 4A) and in the angiogenic endometrium from mice in estrus (Figure 4B, lower panels). On the contrary, the vessels in all other tissues analyzed including the heart, lung, kidney, intestine, as well as normal endometrium were not stained (data not shown, and Figure 4B, upper panels). These results are consistent with the CD13 expression pathway that is upregulated in endothelial cells during angiogenesis [45]. 

### 2.5. Characterization of the Signaling Properties of NGR-hTNF

**Figure 5:**
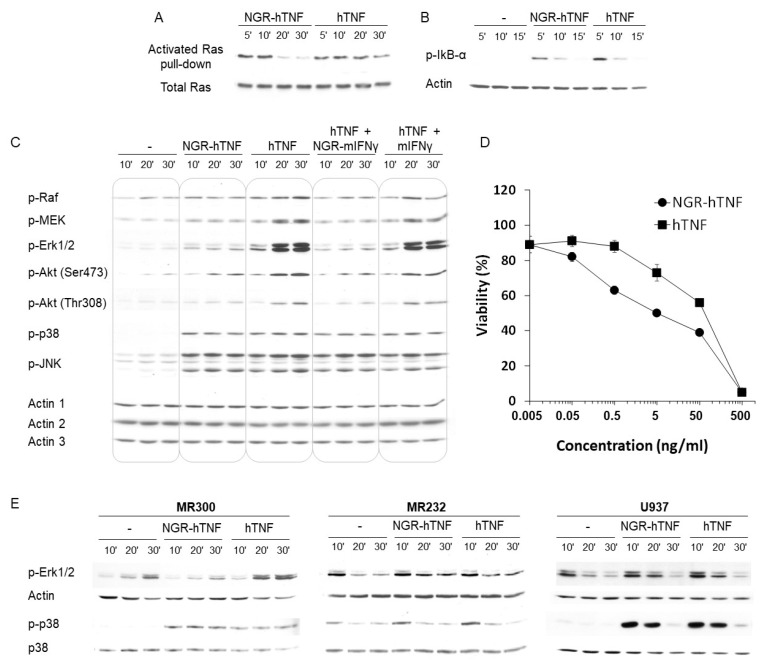
NGR-hTNF and hTNF cell signaling in CNGRC-binding and CNGRC-non-binding cell lines. (**A**) MR300 cells were stimulated with NGR-hTNF or hTNF and Ras GTPase activation was evaluated. Total Ras blot was performed for normalization. A representative experiment out of two is shown. (**B**) MR300 cells, untreated or incubated with NGR-hTNF or hTNF were analyzed for IKB-α phosphorylation, indicative of active NF-κB nuclear translocation [49]. Actin blot was performed as loading control. A representative experiment out of three is shown. (**C**) MR300 cells were left untreated or incubated with NGR-hTNF, hTNF alone or in combination with either NGR-mIFNγ or mIFNγ, and their lysates were analyzed for phosphorylation of the indicated kinases. Actin 1 is the loading control for Raf, MEK, and Akt (Ser473) blots; actin 2 is the loading control for Erk and Akt (Thr308) blots; actin 3 is the loading control for p38 and JNK blots. A representative experiment out of three is shown. (**D**) Cytotoxicity of NGR-hTNF and hTNF was tested on MR300 as described in Section 4. One representative experiment out of three is shown (mean ± SE). (**E**) CNGRC-binder cells (MR300) and CNGRC-non-binder cells (MR232 and U937) were incubated with NGR-hTNF or hTNF and analyzed for Erk1/2 or p38 phosphorylation. Blotting with actin or p38 was performed, after stripping, as the loading control. One representative experiment out of two is shown.

To investigate whether the interaction of the CNGRC peptide with CD13 has a role in the signaling properties of NGR-hTNF, the cell signaling triggered by NGR-hTNF and hTNF in the MR300 cell line were compared. A preliminary analysis, conducted on 33 phosphoproteins by Kinexus Bioinformatics Corporation (CAN), revealed that only three proteins were differently phosphorylated when the cells were treated with NGR-hTNF in comparison to hTNF: Erk1 (−36%), Erk2 (−35%), and Akt-Ser473 (−36%). The further characterization of the NGR-hTNF cell signaling, as shown in Figure 5 panels A, B and C (first nine columns), showed that Ras, NF-κB, and the whole Raf/MEK/Erk pathway were less activated by NGR-hTNF as compared with hTNF. Moreover, Akt phosphorylation was impaired on both Ser473 and Thr308. The reduction of IKB-α phosphorylation induced by NGR-hTNF compared with hTNF was moderate but reproducible. Interestingly, p38 and JNK kinases were activated to the same extent by NGR-hTNF and hTNF (Figure 5C), strongly suggesting that the inhibitory effects induced on MAPK and Akt pathways were not due to the different affinities of NGR-hTNF and hTNF. This hypothesis was confirmed by the observation that a major and specific inhibition of the Raf/MEK/Erk and Akt pathways was detected with the co-administration of hTNF and a NGR-murine (m)IFNγ protein (Figure 5C, columns 10–12). Since mIFNγ is species-specific and does not bind hIFNγ receptors [46], the observed activity of NGR-mIFNγ can be mediated only by the CNGRC peptide. As expected, the hTNF signaling was not modified by mIFNγ (Figure 5C, columns 13–15). Altogether, these data indicate that the CNGRC targeting peptide, through the binding to CD13, interferes with the signaling originated from the TNFR engaged by hTNF. Since transient versus sustained ERK activation has been linked to different cellular responses [47,48] this study investigated whether the signal transduction perturbation induced by NGR-hTNF could lead to the modulation of a biological effect. NGR-hTNF and hTNF cytotoxic activity was tested on MR300 cells, in vitro (Figure 5D). In agreement with the reduced activation of the Ras/Raf/MEK/Erk and Akt pro-survival pathways observed with NGR-hTNF, it was found that viability was reduced (*p* < 0.05) in the cells pulsed with the targeted cytokine in comparison with hTNF.

Next, this study, comparing the Erk1/2 phosphorylation in CNGRC-binder and non-binder cell lines, confirmed the requirement for the CNGRC-binding CD13 isoform in the signaling modulation induced by NGR-hTNF. As shown in Figure 5E, NGR-hTNF reduced Erk1/2 activation, compared to hTNF, only in cells expressing the CD13 isoform that binds the CNGRC peptide (MR300). In the CNGRC non-binder cell (MR232 and U937), NGR-hTNF and hTNF phosphorylate Erk1/2 to the same level. This shows that the CNGRC peptide per se, in the absence of its receptor (the specific CD13 isoform), does not modify hTNF activity. Phosphorylation of p38, as previously observed, was induced similarly by NGR-hTNF and hTNF.

All together, these results strongly suggested that, in CNGRC-binding cells, the signaling induced by NGR-hTNF is mediated by both CD13 and TNFR, and correlated with an increased cytotoxic activity.

### 2.6. NGR-hTNF Signal Transduction Pathways and Biological Effects in HUVEC

**Figure 6:**
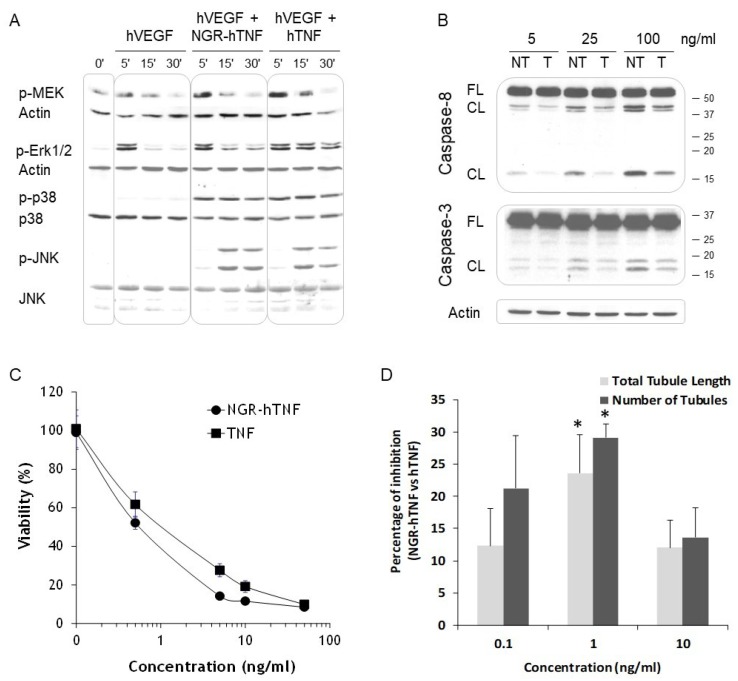
Cell signaling and cytotoxicity of NGR-hTNF and hTNF, in HUVEC. (**A**) Starved HUVEC cells were stimulated with NGR-hTNF or hTNF in the presence of hVEGF (as described in Section 4), and their lysates analyzed for phosphorylation of the reported kinases. Actin, p38 and JNK blots were performed, after stripping, as a loading control. A representative experiment out of three is shown. (**B**) Starved cells were incubated for 30 min with NGR-hTNF (NT) or hTNF (T) and analyzed after 4 h. The fragmentation of Caspases 8 and 3 was detected with antibodies that recognize the full length (FL) and the cleaved (CL) forms. Actin blot was performed as a loading control. Molecular mass markers (kDa) are shown to the right of the blots. One representative experiment out of two is shown. (**C**) Cytotoxicity of wild type and recombinant hTNF was tested on HUVEC as described in Materials and Methods. One representative experiment out of five is shown (mean ± SE). (**D**) Comparison between NGR-hTNF and hTNF effects on capillary-like structures formation by HUVEC was performed with an in vitro test (as described in Section 4). At day 10, the cells were stained with an anti-CD31 Ab and tubule images analyzed for the quantification of total tubule length, and the number of tubules. The results are expressed as a percentage of the decrease induced by NGR-hTNF over hTNF. A representative experiment out of two is shown (mean ± SE; * *p* < 0.05).

Next, the NGR-hTNF signal transduction pathways and eventual biological effects in HUVEC cells were investigated. Under the experimental condition used (data not shown), the activation of the Raf/MEK/Erk pathway induced by hTNF was detectable only if the cells were treated simultaneously with VEGF, a growth factor produced in neoangiogenic vessels [50]. As previously found with other CNGRC-binder cells, it was observed that, also in HUVEC, NGR-hTNF activated MEK and Erk in a less sustained way compared to hTNF, while p38 and JNK were activated to the same extent by both cytokines (Figure 6A). In HUVEC, the differences in MEK and Erk activation were less marked than in MR300 but consistent, as proved by the quantification of three independent experiments shown in Supplementary Figure S4. The moderate changes observed constitute a limitation of the study probably related to the in vitro use of primary HUVEC cells and not of tumoral neoangiogenic cells, which are the in vivo specific target of the NGR-peptide (Figure 4A).

This study then investigated if the perturbation in cell signaling induced by NGR-hTNF led to a modulation of its biological effects. It is known that hTNF induces apoptotic cell death by caspases activation mediated by the cleavage of full-length Caspase 8 and 3 in the active cleaved forms. The fragmentation of Caspases 8 and 3 was higher in HUVEC treated with NGR-hTNF, as compared with the cells treated with hTNF, at the three tested concentrations (Figure 6B). Related to this increase in caspase activation, it was found that viability was significantly reduced in the cells pulsed with NGR-hTNF in comparison with hTNF (Figure 6C), with a significant EC50 reduction (*p* < 0.05; 5 experiments). These results are in agreement with the high level of NGR-TNF-mediated apoptosis detected in vivo, as already described in the literature [7,8], and shown in Supplementary Figure S5A. The apoptosis of endothelial cells along tumor vessels was detected in histological sections of CT26 tumor harvested from tumor-bearing mice 24 h after NGR-hTNF administration, but not after hTNF treatment.

Since NGR-hTNF targets neoangiogenic vessels, its activity was next assessed in an in vitro angiogenesis assay that allows the analysis of tubule-like structures formation. HUVEC were pulsed with different concentrations of NGR-hTNF, that induced a reduction in the total tubule length, and the number of tubules compared to hTNF (Figure 6D and Supplementary Figure S5B). 

Overall, these results suggested that the CNGRC binding to CD13 not only determines the homing of NGR-hTNF to tumor vessels, but also a reduction of pro-survival pathways and an increased activation of caspases, thus supporting the increased biological activity of NGR-hTNF observed in vitro.

## 3. Discussion

As reported for other targeted drugs, NGR-hTNF has an increased activity, compared to the wild type cytokine, because the CNGRC peptide mediates the targeting to the neoangiogenic tumor vessel [2,51]. Overall, the results shown in this paper shed some light on additional mechanisms underlying the antitumor activity of NGR-hTNF. First, by means of crystallography and a binding kinetic analysis performed with NGR-hTNF, this study demonstrated that the presence of the CNGRC peptide does not alter neither the conformation of the hTNF molecule, nor its kinetic behavior in the binding of both TNFR1 and TNFR2. Moreover, when NGR-hTNF interacts with the TNFR, the N-terminal CNGRC peptide is characterized by high flexibility and remains exposed for the interaction with other receptors. Second, it was confirmed that the CNGRC motif binds to a specific isoform of CD13, whose expression is restricted to neoangiogenic cells characteristic of the tumor vasculature and to some tumor cell lines. Further, it was demonstrated that this specific interaction with CD13 increased the overall NGR-hTNF binding affinity to target cells. By investigating the signal transduction triggered by NGR-hTNF in CD13 positive cells, this study found that NGR-hTNF signals differently from hTNF, inducing an impaired activation of several pro-survival pathways (Erk, Akt, NF-κB), without affecting other pathways related to stress and cell death (p38 and JNK). This new signaling property was not due to the increased binding affinity of NGR-hTNF to the target cells. Indeed, a CNGRC peptide carried by a neutral carrier (NGR-mIFNγ) interferes with the cell signaling triggered by hTNF, with a pattern identical to the signaling of NGR-hTNF. This data suggests that the CD13 engagement has an active role in the impairment of the TNFR signaling. This hypothesis was further supported by the observation that the CNGRC motif had a modulatory activity on Erk activation induced by two other cytokines, IFNγ and IL12p70, whose NGR-tagged form showed an increased activity at low doses, in vivo, in murine tumor models ([52] and International Patent Application N.O. WO2008/152508, Applicant Molmed SpA). Indeed, both IFNγ-NGR and IL12p70-NGR specifically triggered a reduced Erk1/2 activation, in comparison with the respective non-targeted cytokines, with no effect on other cell signaling proteins (Supplementary Figure S6). 

All together, these results indicate that the CNGRC peptide, along with the tumor-homing properties, also exerts a direct effect on the intracellular signaling pathway through the binding to CD13. This data is of particular relevance since the CNGRC-motif has been used by many investigators as a mere tumor-homing peptide to deliver imaging and therapeutic agents to angiogenic vessels [53]. 

**Figure 7:**
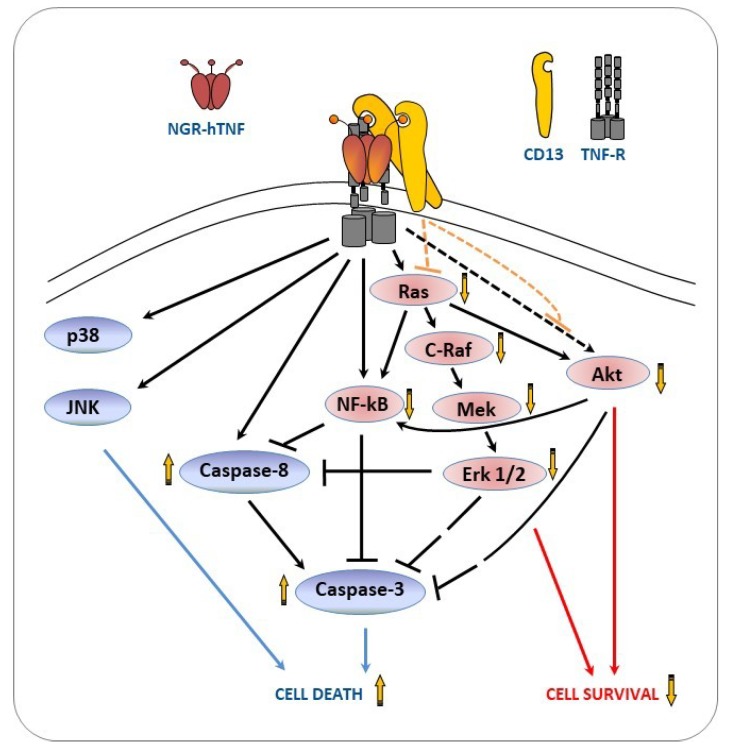
Interaction between TNFR and CD13 cell signaling. The proteins in blue circles are stress or cell death related, while the proteins in orange circles are pro-survival factors. The black lines indicate activator (arrow head) or inhibitor (not arrow head) effects triggered by the TNFR, or the interactions between the target proteins. The orange lines show the interference of the CD13 on the cell signaling induced by TNFR. The dotted lines indicate that the mechanism of action is not fully elucidated. The vertical short orange arrows indicate an increase (upward pointing arrow) or decrease (downward pointing arrow) in the activation induced by NGR-hTNF in comparison with hTNF. The resulting biological activities are indicated by blue (cell death) and red (cell survival) arrowed lines.

The reduced activation of the pro-survival pathways, induced by NGR-hTNF, correlated with an increased caspase activation and with an inhibition of cell survival, compared with hTNF. To summarize the NGR-hTNF signaling, this study reported all the possible interactions between the different signaling players in the flow chart shown in Figure 7. 

As shown, the TNF receptor triggering activates both the cell survival (NF-κB, Erk1/2, and Akt) and stress/death (p38, JNK and caspases) pathways, while the engagement of the CD13 receptor impairs the pro-survival pathways. The CD13-drived impairment of Ras activity may account for the reduced activation of NF-κB, Erk1/2 and Akt, which, in turn, may increase cell death through a distinct mechanism. First, these three signal-proteins are direct or indirect negative regulators of the initiator caspases 8 and/or the effector caspase-3 [23,25,27,54,55]. Second, Erk1/2 and, especially Akt, may block apoptosis indirectly activating several pro-survival components (reviewed in [56,57]). 

To explain how CD13 engagement by NGR-hTNF interferes with the signaling triggered by the TNFR, two hypotheses can be formulated, based on the literature. CD13 may act directly on the intracellular signaling pathways through the involvement of auxiliary proteins [37], or it may act indirectly on the cell signaling, through plasma membrane organization, and the modulation of the cellular localization/activation of cytokine receptors [36]. To understand which mechanism is involved, further studies are needed. Overall, NGR-hTNF, through the impairment of NF-κB, Erk1/2 and Akt activation, decreases cell survival and increases the pro-death state promoted by caspases and JNK [58,59]. A decreased tubule formation was also observed in the presence of NGR-hTNF respect to hTNF, by an in vitro assay in which endothelial cells were co-cultured with primary human interstitial cells. Although this assay gives information more representative of capillary formation than other tests as tubule-like formation on supportive matrix [60], our results need to be confirmed by other assays such as, spheroid sprouting or in vivo angiogenesis assays.

The increased cytotoxicity observed for NGR-hTNF, in vitro, correlates with the detection in the in vivo murine models of a marked caspase activation of neoangiogenic endothelial cells that through the direct inhibition of tubules formation, or through the induced normalization of the tumor vasculature and the involvement of the immune system, eventually leads to the inhibition of tumor growth. Furthermore, in humans, a better clinical outcome was observed in patients with elevated blood markers of angiogenesis and of the immune response. Indeed, in a subset of mesothelioma patients treated with NGR-hTNF, the improved clinical outcomes have been related to an increased tumor hypoxia/angiogenesis, evaluated as high baseline serum levels of lactate dehydrogenase [11]. Moreover, high baseline blood lymphocyte counts have been found to predict improved outcomes after the administration of NGR-hTNF combined with chemotherapy [61]. These clinical results are in line with the hypothesized drug’s mechanism of action, which is based on an improved intratumoral penetration of chemotherapeutic agents and increased tumor lymphocyte infiltration, due to NGR-hTNF activity on newly formed tumor vasculature.

In summary, the evidence that NGR-hTNF, in vitro, has a distinctive signaling pattern and increased biological effects, strongly supports the hypothesis that the antitumor activity of NGR-hTNF is improved, in vivo, not only by the targeted delivery to neoangiogenic vessels, but also because of its peculiar mechanism of action. 

## 4. Materials and Methods 

### 4.1. Cell Lines

Human lung cancer (MR300 and MR232 [62], human melanoma (MSR3, and GR4-mel) [63], and pro-monocytic human myeloid leukaemia (U937) cells were cultured in RPMI medium (Lonza, Basel, CH, USA) supplemented with 10% fetal bovine serum (FBS, heat inactivated; HyClone, Logan, UT, USA), 2 mM glutamine, 100 units/mL penicillin and streptomycin (Gibco, Gaithersburg, MD, USA). Human umbilical vein endothelial cells (HUVECs; Promocell, Heidelberg, DE, USA) were grown in complete EGM-2 (Lonza). Mouse fibroblast cells (L-M) were grown in complete IMDM medium (Lonza). Human muscle-derived perivascular stem cells (Mesoangioblasts) were derived, as previously described [42], from a skeletal muscle fragment (Biopredic International, Saint-Grègoire, France). Mesoangioblasts were cultured in IMDM supplemented with 10% FBS, 10,000 U/mL penicillin, 10 μg/mL streptomycin, 2 mM glutamine, 5 ng/mL human bFGF (PeproTech, London, UK), 1% non-essential aminoacids (Sigma Aldrich, St. Louis, MO, USA) and 0.1 mM β-mercaptoethanol (Gibco, Gaithersburg, MD, USA), at 3% O_2_. Mesoangioblasts were characterized by an immunophenotypic analysis and found, as expected, CD13+/CD44+/NG2+/CD56−/CD31−/CD34−/CD45−. CD13 silencing was performed with lentiviral vector (pLKODpuroshCD13ΔNGFR#7, Sigma Aldrich, St. Louis, MO, USA). VSV-G pseudotyped lentiviral vector stocks were used to transduce mesoangioblasts in the presence of polybrene. The cells with more than 97% of silencing were used for the experiments.

### 4.2. Animal Models

CT26 colon carcinoma cells, a *N*-nitroso-*N*-methylurethane-(NNMU)-induced, undifferentiated colon carcinoma cell line (ATCC, Manassas, VA, USA), were cultured in RPMI supplemented with 10 mM Hepes, 1 mM sodium pyruvate, 4.5 g/L glucose, 1.5 g/L sodium bicarbonate, 2 mM l-glutamine and 10% FBS (Hyclone, Logan, UT, USA). CT26 cells (1 × 10^5^) were s.c. injected into syngeneic BALB/c mice (Charles River, Wilmington. MA, USA). The in vivo experiments were performed when the tumour sizes reached 500 mm^3^. The in vivo experiments meet the standards required by the UKCCCR guidelines (Workman et al, 2010). The experimental protocols were approved by the Institutional Animal Care and Use Committee of San Raffaele Scientific Institute (project identification code: IACUC 492; date of approval: 2011).

### 4.3. Cytokines

hTNF, hIFN-γ, and mIFN-γ were purchased from PeproTech (London, UK). NGR-hTNF [11] was obtained by genetic engineering technology, coupling the peptide CNGRCG to the amino terminus of the wild type cytokine [2]. hTNF and NGR-hTNF cytotoxicity was tested on a CD13 negative, NGR-non-binder cell line (L-M cells), as described under Cytotoxicity assay, and showed always identical specific activity. hIFN-γ-NGR and NGR-mIFN-γ were also obtained by genetic engineering technology, as previously described [52]. IL12p70 and IL12p70-NGR were produced as described in the International Patent Application n.o. WO2008/152508, (Applicant Molmed SpA). IL12p70 and IL12p70-NGR induction of hIFN-γ was evaluated on human peripheral blood lymphocytes (PBL), by ELISA according to the manufacturer’s instructions (BD Bioscience, San Jose, CA, USA). The wild-type and targeted cytokines showed always identical specific activity.

### 4.4. Peptide Binding Assay

The binding assay of CNGRC and CisoDGRC biotinylated peptides, to adherent cells was carried out as follow. The cells, grown in chamber slides (5 × 10^4^ cells/well, plated 48 h before the experiment), were washed in binding buffer (25 mM Hepes buffer, pH 7.4, containing 150 mM sodium chloride, 1 mM magnesium chloride, 1mM manganese chloride, 1% bovine serum albumin (BSA), and 0.1% sodium azide), and incubated with the indicated Biotinylated peptides (1:100 dilution in binding buffer) or the CD13 antibody (anti CD13-PE, WM15 clone, 1:150 diluition) for 1 h at room temperature. After washing with the binding buffer, the cells were incubated with a secondary anti-biotin antibody for 30 min at room temperature and washed again. The cells were fixed with 2% paraformaldehyde, 3% sucrose in PBS for 15 min at room temperature. The DNA was counterstained with DAPI (Invitrogen, Life Technologies Ltd., Carlsbad, CA, USA), and the images were acquired using an Olympus BX61 microscope (excitation filter, E460SPUVv2; emission filter, D605/20 nm, Chroma Technology Corp., Bellows Falls, VT, USA) and then analysed using ImageJ (NHI). The data were expressed as an arbitrary unit, which is the ratio between the pixel of the specific staining positive area and the pixel of the DAPI positive area.

### 4.5. Conjugation of Peptide to Nanoparticles (Qdots)

The quantum dots were prepared as previously described [41]. Briefly, new amine-modified Qdot nanoparticles (Qdot605 ITK Amino (PEG) Quantum Dots; Invitrogen, Life Technologies Ltd.) were activated with the bis[sulfosuccinimidyl] suberate crosslinker (Pierce, Waltham, MA, USA), according to the manufacturer’s instructions. The *N*-hydroxysuccinimide-nanoparticles were purified from an unreacted crosslinker by gel-filtration chromatography on NAP-5 column (GE Healthcare, Chicago, IL, USA). Equal amounts of the colored fractions (half vial of Qdots) were incubated in 32 μL of water alone or containing 160μg of AcCNGRCG-VRSSSRTPSDYk (AcNGR-hTNF1-11), or AcCisoDGRCG-VRSSSRTPSDYk (_ac_isoDGR-hTNF1-11), for 2 h at room temperature. The conjugates (called Qdot, NGR-Qdot, and isoDGR-Qdot, respectively) were then separated from the free peptides by ultrafiltration (Ultra-4 Ultracel-100K; AMICON, Millipore, Burlington, MA, USA), re-suspended in 25 mmol/L Hepes, 150 mmol/L NaCl in sterile water, and stored at 4 °C.

### 4.6. In Vivo Administration of Peptid-Qdots and Whole Mount Analysis

The NGR-Qdot or Qdot were diluted 1:10 in 200 μL of saline and injected intravenously in mice bearing palpable and not necrotic subcutaneous CT26 tumors or in female mice in estrus. After 10 min of circulation, the mice were perfused (3 mL/min) with saline for 5 min, then the tumor or endometrium was collected. The analysis of the tumor tissues in the whole mount was performed after incubation with Alexa488-anti-CD31 mAb (Invitrogen, Lifetechnologies Ltd., Carlsbad, CA, USA) that specifically stained the blood vessels. Briefly, the tumors were minced in small pieces and placed into a 24 well plate. The samples were blocked using Fc Block (BD Biosciences, San Jose, CA, USA) at 10 μg/mL in 200 μL of PBA (phosphate buffered saline + 1% BSA + 0.1% sodium azide) with shacking at 4 °C for 10 min. Anti-CD31 was added directly to the wells (1:30 dilution), and incubated with shaking at 4 °C for 2 h. The samples were then washed four times with 1ml of PBA at 4 °C for 30 min. The stained samples were analysed by placing the tumors on a glass microscope slide (VWR Scientific, Radnor, PA, USA) with two drops of PBA. A coverslip was placed on top of the slices and gently pressed down. The slides were then analyzed at the fluorescent microscope (Olympus BX61 microscope; excitation filter, E460SPUVv2; emission filter, D605/20nm, or U-MNIB BP470-490, Chroma Technology Corp., Bellows Falls, VT, USA).

### 4.7. TNF Binding Assays and Competition

The binding assays of hTNF to adherent cells was carried out as follows. NGR-binder (MR300) and NGR-non-binder cells (GR4-mel cells) were grown in chamber slides (5 × 10^4^ cells/well, plated 48 h before the experiment). After washing in binding buffer (25 mM Hepes buffer, pH 7.4, containing 150 mM sodium chloride, 1mM magnesium chloride, 1mM manganese chloride, 1% BSA, and 0.1% sodium azide), the cells were incubated with TNF-Fc (1:50 dilution in Binding Buffer; Alexis, San Diego, CA, USA) for 1 hour at room temperature, and then with the secondary Alexa-594-conjugated goat anti-human antibody (1:70 dilution, Invitrogen, Lifetechnologies Ltd., Carlsbad, CA, USA). After washing with binding buffer, the cells were fixed with 2% paraformaldehyde, 3% sucrose in PBS for 15 min at room temperature. The DNA was counterstained with DAPI (Invitrogen, Life Technologies Ltd.), and the cells were then analyzed using an Olympus BX61 microscope (excitation filter, E460SPUVv2; emission filter, D605/20 nm, Chroma Technology Corp.). For the competition experiments, the hTNF-Fc staining was performed as described above in the presence of various amounts (from 0.01 to 30 µg/mL) of either NGR-hTNF or h-TNF on MR300 cells. In order to define the role of the NGR portion of NGR-hTNF in the competition experiments, the staining of hTNF-Fc plus NGR-hTNF, or hTNF (at the concentrations that give maximum displacement of hTNF-Fc) was competed by various amounts (from 19 to 500 µg/mL) of CNGRC-GVRY (NGR) or unrelated CSGRC-GVRY (SGR) competitor peptides. The images were acquired with CellF Software (Olympus Soft Imaging Solutions GmbH) and the quantification of staining intensity was performed by using ImageJ (NIH). At least 5 pictures (magnification 200×) were analyzed per each well. The results were expressed as the percentage of the control (sample incubated with TNF-Fc alone). All the experiments were repeated at least three-times. 

### 4.8. Flow Cytometry

Exponentially growing cells, detached using enzyme free dissociation buffer (Accutase, Invitrogen, Lifetechnologies Ltd., Carlsbad, CA, USA) if growing in adherence, were incubated with anti-human CD13-PE Ab (clone WM15; BD Bioscience, San Jose, CA, USA) or control isotype Ab, for 30 min at 4 °C, washed and analyzed by flow cytometry. Fluorescence was measured using FacsCalibur (BD Biosciences) and analyzed using FloJo (FlowJo) software.

### 4.9. X-ray Diffraction

The recombinant NGR-hTNF was produced as previously described [2] in Escherichia coli cells, and purified in native conditions through ammonium sulfate precipitation followed by hydrophobic interaction and size exclusion chromatography. The tetragonal NGR-hTNF crystals were obtained using the hanging drop diffusion method from 2.8 M MgSO_4_, 1 M NaCl, and 0.1 M MES at pH 6.0, and the diffraction quality crystals were grown through streak- and macroseeding. X-ray diffraction datasets to 2.5 Å resolution were collected at 100 K at the ID14-3 beamline of the ESRF (Grenoble, F). The data were integrated and reduced using the programs XDS and AIMLESS. The initial phases were obtained using the molecular replacement method with the human TNFα structure determined in a complex with a camelid antibody (PDB ID 5M2J) as a search model. After rigid body refinement, the structure was rebuilt using the program AutoBuild, and refined in cycles of manual adjustment into electron density maps using the program Coot [64], and restrained refinement with a maximum likelihood target function with Phenix [65]. The model stereochemistry was monitored continuously with the program Molprobity [66]. The integrated dataset included all the reflections with CC_1/2_ > 0.3 in the highest resolution shell, and a paired refinement procedure was used to assess the maximum resolution of the data yielding an improved model, as judged from the lowest divergence between R_crys_ and R_free_ [67]. The data collection and refinement statistics are presented in Table 1. The integrated diffraction data and the refined NGR-hTNF coordinates were deposited with the PDB, accession code 6RMJ.

### 4.10. 2D TR-NOE Spectroscopy (TR-NOESY) Experiments by NMR Technique

This study investigated the binding of CNGRC cyclopeptide, and the control cyclopeptides (CARGC, CDGRC) onto living cell lines by TR-NOESY, as previously described [43]. Briefly, the cells were detached in DPBS (Lonza) with 2 mM EDTA, and transferred into a 3 mm NMR tube at the concentration of 8–10 × 10^6^ cells /200 μL of deuterated DPBS supplemented with 3 mM MgCl_2_. The NMR experiments were performed at 37 °C, for 2 h, at a field strength of 600 MHz. The cell suspension homogeneity and >75% cell viability were achieved throughout each experiment. To discriminate binder from non-binder cells, the different NOE build-up rate, i.e., the time required to achieve maximum NOE intensity, was exploited, and the experiments were performed at short (100 ms) and long (600 ms) mixing times. The ligands interacting with surface receptors have a fast NOE build-up, with the maximum NOE intensity (cross-peaks with the same sign as the diagonal) at short mixing time (100 ms), and at longer mixing time (600 ms) the signal has already decayed. Conversely, the negative control peptides that do not bind and therefore have a slower NOE build-up curve, have no (or very weakly positive) NOE effect at 100 ms, whereas they develop the strongest NOE intensity (cross-peaks with opposite sign with respect to the diagonal) at longer mixing time (600 ms). 

### 4.11. Surface Plasmon Resonance Analysis

The interactions between NGR-hTNF or hTNF and TNFRs were determined using surface plasmon resonance approaches as previously described [68], with a Biacore T-100 (GE Healthcare). Briefly, TNFR1 and TNFR2, fused to the Fc portion of an IgG molecule, were cross-linked to an anti-Fc Ab covalently immobilized to the CM5 chip surface, and exposed to different concentrations of NGR-hTNF or hTNF. Due to a very low dissociation of hTNF from the receptors, as a first approach of analysis, the kinetic titration [69] was selected, which allowed to perform sequential injections of a TNF concentration series without any regeneration steps. This approach provided qualitative indications about the kinetic of the interaction but did not allow the description of complex interactions. Next, a conventional standard kinetic, which involves injections of several concentrations of analyte over the same ligand surface, and regeneration of the surface between binding cycles (step with Gly-HCl pH2), was applied. Since it has been demonstrated, using different types of sensor chips, that the stoichiometry of the cytokine-receptor complex is influenced by the receptor density on the surface [70], the antibody (aminecoupling: EDC/NHS and Ethanolamine injections) has been covalently immobilized on the sensor surface of different chips (C1, CM4 and CM5). A stoichiometry evaluation at different receptors densities and a complete standard kinetic analysis of NGR-hTNF or hTNF interaction with TNFRs, in order to fit the data with different models (1:1, 1:2 and 1:3), was performed. The different concentrations (0.6–83 nM) of NGR-hTNF or hTNF were injected in duplicates over TNFR1 and TNFR2 at a level of 200RU (medium immobilization level that gives a workable signal). The optimal reproducibility between the replicates has been obtained. The sensorgrams obtained with standard kinetic were analyzed using three different models: 1:1, 1:2 (Bivalent equation: from BiaEvaluation software 4.1) and 1:3 (Trivalent equation: from [71]). For TNFR1, based on the statistical parameter (chi2), it was confirmed that the data can be better fitted with a complex model, but it was not possible to discriminate between 1:2 or 1:3 models (as expected from the stoichiometry data). In order to avoid the over-fitting issues derived from the high complexity of trivalent equation, the data of 1:2 model was reported.

### 4.12. Analysis of Signal Transduction

For the analysis of signal transduction, the cells were starved in RPMI supplemented with 0.1% BSA (Sigma, MO, USA), from overnight (U937) to 2 days (MR300, MR232), and then stimulated with the indicated cytokines in the absence or presence of 10% serum. HUVEC were grown in a regular medium for two days, starved overnight in EBM medium (Lonza) supplemented with 0.1% BSA (Sigma, St. Louis, MO, USA), and then stimulated with the indicated cytokines in the absence or presence of hVEGF (20 ng/mL; hVEGF-A165, PeproTech, London, UK). NGR-hTNF and hTNF were added at concentrations ranging from 500 ng/mL (all the experiments shown) to 5 ng/ml (data not shown). NGR-mIFNγ and mIFNγ, were used at 1 µg/mL. At the indicated time points, the cells were put on ice, washed twice with ice-cold PBS, and lysed in lysis buffer T (50 mM HEPES, 150 mM NaCl, 1.5 mM MgCl_2_, 1 mM EGTA, 1 mM EDTA, 10% Glycerol, and 1% Triton X-100), supplemented with fresh proteases inhibitors cocktail (CØmplete, Roche, CH, USA), 5 µM pepstatin, 1 mM sodium othovanadate, and phosphatase inhibitor cocktail (PhosStop, Roche, CH, USA). Equal amounts of total protein were subjected to SDS-PAGE and transferred to 0.4 μm nitrocellulose membranes (Schleicher & Schuell, BioScience, Dassel, DE, USA). The filters were blocked in TBST (0.1% Tween in TBS) containing 5% non-fat dry milk (Merck Millipore, Burlington, MA, USA) for 1 h, at RT, and then immunoblotted with the indicated primary Ab, following the manufacturer’s instruction, overnight, at 4 °C. The blots were then incubated with the appropriate secondary horseradish peroxidase (HRP)-conjugated antibodies (GE Healthcare, Chicago, IL, USA), and developed using the ECL system (GE Healthcare). On the same membrane, after stripping, more than one immunoblot was performed. Each membrane was immunoblotted first with the anti-phosphorylated kinases Abs, and last with anti-actin Ab. The following anti-phospho-specific antibodies were purchased from Cell Signaling Technology (Danvers, MA, USA): anti-phospho Raf (Ser338) Ab, anti-phospho-MEK1/2 (Ser217/221) Ab, anti-phospho-Erk1/2 (Thr202/Tyr204) Ab, anti-phospho-Akt (Ser473) Ab, anti-phospho-Akt (Thr308) Ab, anti-phospho-p38 MAP Kinase (Thr180/Tyr182) Ab, anti-phospho-JNK (Thr183/Tyr185) Ab, anti-phospho-IκB-α (Ser32) Ab. Anti phospho-Stat-1 (Tyr701) Ab was purchased from Millipore (Burlington, MA, USA). The antibodies against the corresponding not phosphorylated proteins were purchased from Cell Signaling Technology (MA, USA). Anti-actin (Sigma-Aldrich, St. Louis, MO, USA) immunoblot was performed on the filters as the loading control. The activation analysis of a large number of proteins was performed by Kinexus Bioinformatics Corporation (CAN), using the Kinetworks phospho-site broad coverage pathway screen (catalog number KPSS 1.3). Briefly, MR300 starved cells were stimulated with NGR-hTNF or TNF (5 ng/mL) and lysed after 10 and 20 min of incubation following the manufacturer’s instructions. Lysates were sent to Kinexus, were thirty-eight phosphorylation sites in 33 phosphoproteins were examined with antibodies that recognize phosphorylated epitopes. To study Ras small GTPase activation, the Active RasPull-Down and Detection Kit (Thermo Scientific, Waltham, MA, USA) were used, following the manufacturer’s instruction.

### 4.13. Caspase Activation and Cytotoxicity Assay

Following the standard procedure [17], caspase activion was detected in HUVEC cells incubated with NGR-hTNF or hTNF, in the presence of 1.5 µg/mL cycloheximide (Sigma-Aldrich), for 30 min. Then, the cells were washed and incubated in the presence of 1.5 µg/mL cycloexamide, without cytokines, for additional 4 h. After two washes with ice-cold PBS, the cells were lysed, and proteins resolved by SDS-PAGE. The activation of caspase 3 and 8 was detected by immunoblot, using the anti-caspase 3 and 8 (Cell Signaling Technology, Danvers, MA, USA), respectively. The detection of active caspase 3 on histological sections of CT26 tumor collected from mice treated with NGR-hTNF or hTNF (100 pg/mouse), and sacrificed after 24 h, was performed with the anti-Cleaved Caspase-3 antibody (Cell Signaling Technology, Danvers, MA, USA). For cytotoxicity analysis, based on protocols reported in the literature [72], subconfluent cells (HUVEC, MR300 or L-M) were incubated with the indicated cytokines concentrations for four hours, washed and then incubated with regular medium for additional 48 h, in the presence of 1 µg/ml actinomycin (Sigma-Aldrich), and 20 mM lithium chloride. Viability was evaluated by standard MTT colorimetric assay.

### 4.14. Tubule Formation Assay

The in vitro formation of vessel-like structures was studied using the Angiokit (TCS Cellworks, Buckingham, UK), following the manufacturer’s instruction. Briefly, HUVEC were co-cultured with primary human interstitial cells, and incubated at day 0, 3, 6, 8 for two hours with different concentrations of NGR-hTNF and hTNF. The tubules formation was analyzed at day 10 by staining with an anti-CD31 Ab, and incubation with an insoluble substrate to have a permanent visualization of CD31 on the cellular surface. The images were taken using a BX61 microscope (Olympus, Hamburg, DE) with CCD camera and analyzed with the AngioSys software (TCS Cellworks), that led to the quantification of the total tubule length, and the number of tubules.

### 4.15. Statistical Analysis

GraphPad Prism version 5.0b was used to assess the statistical significance of the differences in the mean values (two-tailed student’s *t*-test). The difference between designated groups were considered significant at values of *p* < 0.05.

## Figures and Tables

**Table 1:** Crystallographic data collection and refinement statistics.

**Data Collection**	
Crystal parameters	a = b = 92.011 Å; c = 116.879 Å α = β = γ = 90°
Space group	P4_1_2_1_2
Wavelength (Å)	0.931
Resolution range (Å)	49.33–2.65 (2.78–2.65)
Reflections observed	241,457 (32,201)
Reflections unique	15,203 (1973)
Completeness (%)	100.0 (100.0)
Multiplicity	15.9 (16.3)
<I/σ(I)>	15.6 (1.0)
CC_1/2_	0.999 (0.469)
R_pim_	0.035 (0.905)
**Refinement Statistics**	
Reflections used	15138
R_crys_	0.209
R_free_	0.252
rmsd bonds (Å)	0.003
rmsd angles (°)	0.626
**<B> (Å^2^)**	
Protein	89.8
Solvent	63.4
**Ramachandran Statistics (% of Residues)**	
Favoured	96.7
Allowed	3.3
Disallowed	0

**Table 2:** Association constant and kinetic parameters for NGR-hTNF and hTNF interaction with TNFR1 and TNFR2.

		k_on1_ × 10^5^ (M^−1^·s^−1^)	k_off1_ × 10^−4^ (s^−1^)	t_1/2 1_ (min)	K_D_ (nM)	k_on2_ × 10^−3^ (RU^−1^·s^−1^)	k_off2_ × 10^−3^ (s^−1^)	t_1/2 2_ (min)
TNFR1	NGR-hTNF	4.68 *(1.40)*	15.8 *(1.8)*	7.4 *(0.8)*	3.6 *(1.4)*	4.29 *(0.55)*	1.19 *(0.19)*	9.9 *(1.6)*
	hTNF	2.09 *(0.03)*	9.2 *(0.8)*	12.7 *(1.0)*	4.4 *(0.4)*	2.70 *(0.35)*	1.16 *(0.04)*	10.0 *(0.4)*
TNFR2	NGR-hTNF	5.88 *(0.04)*	336 *(1.4)*	0.3 *(0.0)*	57 *(0.2)*	0.54 *(0.00)*	0.39 *(0.00)*	29.4 *(0.1)*
	hTNF	2.74 *(0.23)*	204 *(46.7)*	0.6 *(0.1)*	74 *(1.1)*	0.55 *(0.01)*	0.30 *(0.02)*	38.5 *(2.3)*

Mean and SD (in parentheses) are shown.

## Supplementary Materials

Supplementary materials can be found at https://www.mdpi.com/1422-0067/20/18/4511/s1.
