# Supplementary material for: Mechanism of Action of the Tumor Vessel Targeting Agent NGR-hTNF: Role of Both NGR Peptide and hTNF in Cell Binding and Signaling
Source: Int J Mol Sci. 2019 Sep 12;20(18):4511. doi: 10.3390/ijms20184511 (PMC6769691; doi:10.3390/ijms20184511)
Supplement: Supplementary file 1 [file ijms-20-04511-s001.pdf]

International Journal of
Molecular Sciences

"Mechanism of Action of the Tumor Vessel Targeting Agent NGR-hTNF: Role of Both NGR Peptide and hTNF in Cell Binding and Signaling"

SUPPLEMENTARY FIGURES

A

Cell line	CD13 expression	Maximum NOE intensity			
		CNGRC		CARGC or CDGRC	
		at100ms with negative NOE*	at 600ms with positive NOE**	at 100ms with negative NOE*	at 600ms with positive NOE**
HUVEC	+	X			X
MB	+	X			X
MR300	+	X			X
MSR3	-		X		X
U937	+		X	nd	nd

B

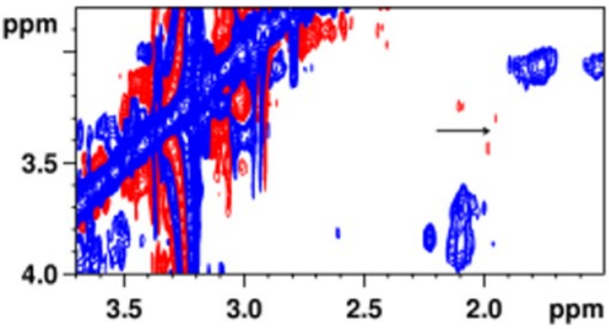

Supplementary Figure 1. TR-NOESY experiments performed on CNGRC or control peptides with viable cells. **(A)** Time and sign of maximum NOE intensity for CNGRC and control peptides (CARGC, CDGRC) on the indicated cell lines. * Ligands interacting with the receptor give the maximum NOE intensity at short mixing time (100ms), with negative NOE correlation. ** Ligands not interacting with the receptor give the maximum NOE intensity at longer mixing time (600ms), with positive NOE correlation. **(B)** When CNGRC peptide binds to the cells (after reaching its maximum intensity at 100ms), at the mixing time of 600ms, the NOE signal has completely decayed (spectrum with MB cells is shown).

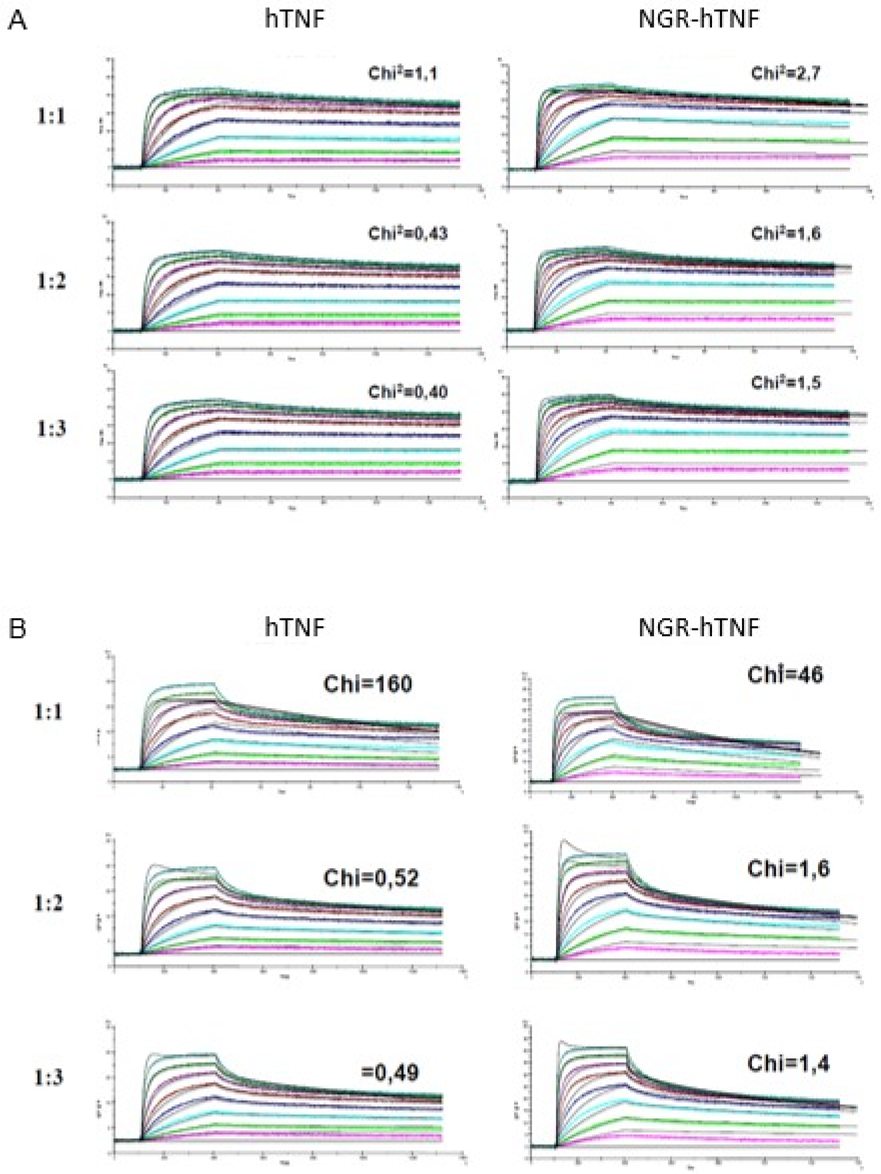

Supplementary Figure 2. Statistical evaluation of different models **(A)** TNFR1. **(B)** TNFR2.

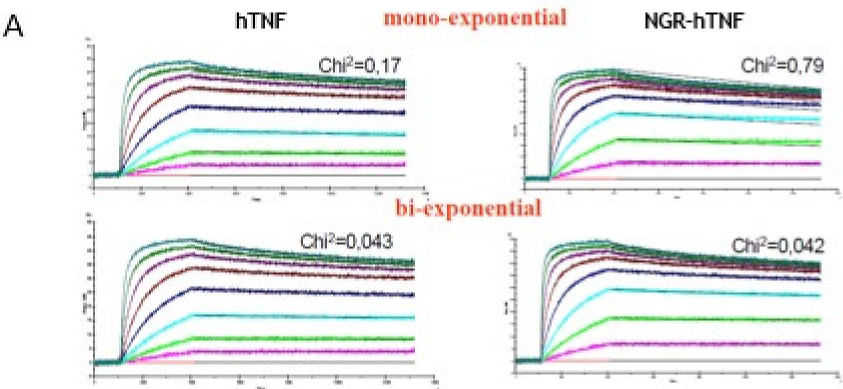

		koff1 (1/s)	t1/2(1) min	koff2 (1/s)	t1/2(2) min
Mono-exp	hTNF	1,56E-04	**74,31**		
		4,95E-06	*2,37*		
	NGR-hTNF	2,07E-04	**56,09**		
		2,12E-05	*5,75*		
Bi-exp	hTNF	2,87E-03	**4,30**	7,46E-05	**185,66**
		1,01E-03	*1,52*	*4,30E-05*	*106,99*
	NGR-hTNF	3,32E-03	**3,68**	7,46E-05	**156,03**
		1,08E-03	*1,20*	*8,84E-06*	*18,50*

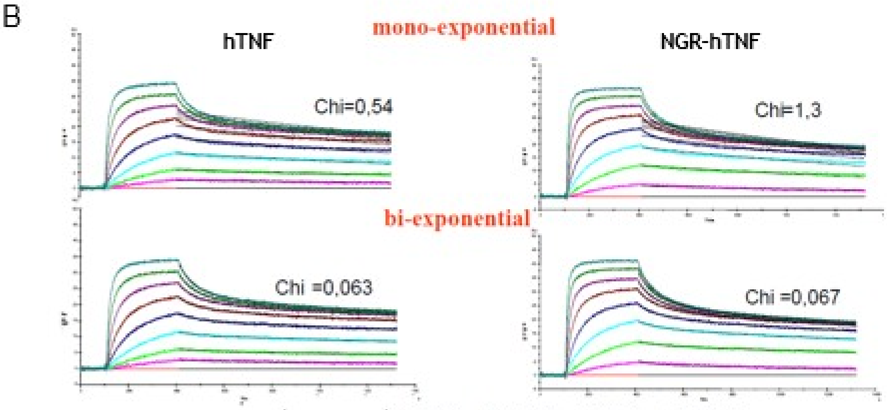

		koff1 (1/s)	t1/2(1) min	koff2 (1/s)	t1/2(2) min
Mono-exp	hTNF	4,20E-04	**27,55**		
		2,55E-05	*1,67*		
	NGR-hTNF	5,71E-04	**20,35**		
		6,22E-05	*2,22*		
Bi-exp	hTNF	1,14E-02	**1,02**	2,55E-04	**45,57**
		9,90E-04	*0,09*	*2,83E-05*	*5,06*
	NGR-hTNF	9,98E-03	**1,17**	3,01E-04	**38,38**
		1,59E-03	*0,19*	*7,07E-06*	*0,90*

Supplementary Figure 3. Dissociation kinetic analysis, statistical evaluation and kinetic parameters from mono- and bi-exponential decay equations. **(A)** TNFR1. **(B)** TNFR2.

Kinase	percent change (NGR-hTF vs hTNF)	
	median	SE
p-MEK	-22	14
p-Erk	-28	8
p-JNK	8	5
p-p38	1.3	5

Supplementary Figure 4. Densitometric analysis of immunoblots was performed to quantify kinases phosphorylation in HUVEC cells. The densitometric values of the phosphorylated bands (arbitrary units) were normalized, and plotted over time. The area under the curve obtained for each kinase was calculated. Results are reported as percent change of NGR-hTNF over hTNF (mean and SE of three independent experiments).

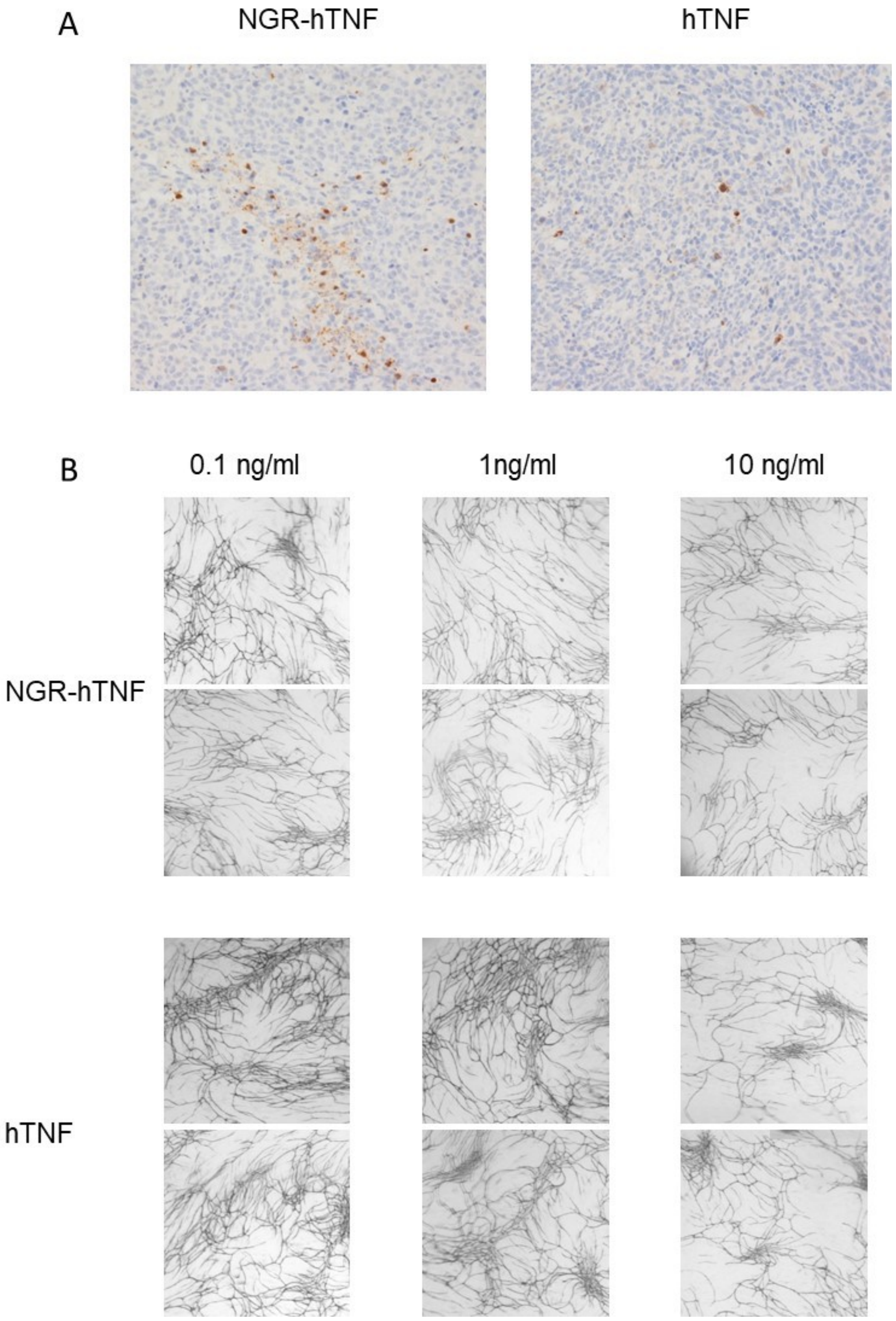

Supplementary Figure 5. NGR-hTNF and hTNF effects on endothelial cells. **(A)** NGR-hTNF induces cell death of tumor vessel endothelial cells, *in vivo*. Mice bearing CT26 colon carcinoma were treated with NGR-hTNF or hTNF (100 pg/mouse), and 24 hours later tumor were collected for analysis of active caspase 3 by using the anti-Cleaved Caspase-3 antibody. As shown in the figure, apoptosis of endothelial cells along the tumor vessels was clearly detected only in histological sections of the tumor treated with NGR-hTNF (cells stained in brown). **(B)** NGR-hTNF inhibits tubule formation, compared to hTNF, on HUVEC cells tested by an *in vitro* co-culture assay. Tubules formation was analyzed at day 10 by staining with an anti-CD31 Ab. Representative images are shown.

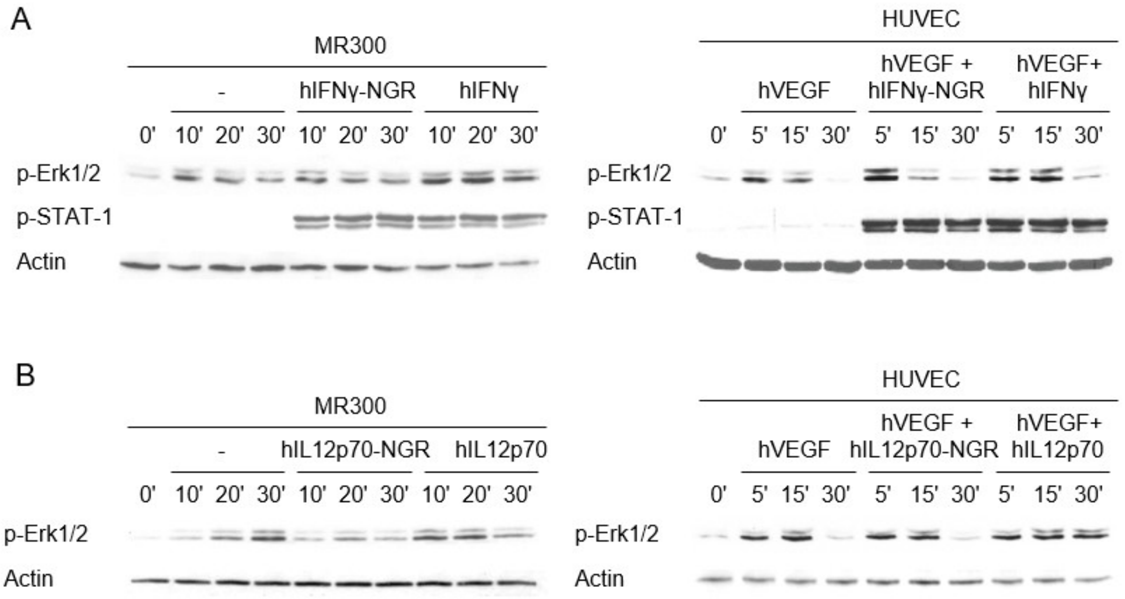

Supplementary Figure 6. NGR-tagged hIFNγ and hIL12p70 signaling. **(A)** Starved MR300 and HUVEC cells were stimulated with hIFNγ-NGR or hIFNγ (both at 1μg/ml), in the presence of 10% serum (MR300) or 20 ng/ml hVEGF (HUVEC). At the indicated time points cells were lysed and proteins analyzed for the phosphorylation of ERK1/2 and STAT-1 by immunoblotting with anti-phospho-specific Abs. Actin was performed on the same filter, after stripping, as normalization control. A representative experiment out of two is shown. **(B)** Starved MR300 cells and HUVEC were stimulated with hIL12p70-NGR or hIL12p70 (both at 2 μg/ml), in the presence of 10% serum (MR300) or 20 ng/ml hVEGF (HUVEC). Cell lysates obtained at the reported times were analyzed for the phosphorylation of ERK1/2 with anti-phospho specific Ab. Actin immunoblot was performed on the same filter, after stripping, as loading control. A representative experiment out of two is shown.
